# Cetuximab and Cisplatin Show Different Combination Effect in Nasopharyngeal Carcinoma Cells Lines via Inactivation of EGFR/AKT Signaling Pathway

**DOI:** 10.1155/2016/7016907

**Published:** 2016-05-24

**Authors:** Jiajia Gu, Li Yin, Jing Wu, Nan Zhang, Teng Huang, Kai Ding, Haixia Cao, Lin Xu, Xia He

**Affiliations:** ^1^The Fourth Clinical School of Nanjing Medical University, Nanjing 210009, China; ^2^Department of Radiation Oncology, Nanjing Medical University Affiliated Jiangsu Cancer Hospital and Jiangsu Institute of Cancer Research, Nanjing 210009, China; ^3^Jiangsu Key Laboratory of Molecular and Translational Cancer Research, Nanjing, China; ^4^Xuzhou Medical College, Xuzhou 210009, China; ^5^Department of Thoracic Surgery, Nanjing Medical University Affiliated Jiangsu Cancer Hospital and Jiangsu Institute of Cancer Research, Nanjing 210009, China

## Abstract

Nasopharyngeal carcinoma (NPC) is a common malignant cancer in South China. Cisplatin is a classical chemotherapeutic employed for NPC treatment. Despite the use of cisplatin-based concurrent chemoradiotherapy, distant failure still confuses clinicians and the outcome of metastatic NPC remains disappointing. Hence, a potent systemic therapy is needed for this cancer. Epidermal growth factor receptor (EGFR) represents a promising new therapeutic target in cancer. We predicted that combining the conventional cytotoxic drug cisplatin with the novel molecular-targeted agent cetuximab demonstrates a strong antitumor effect on NPC cells. In this study, we selected HNE1 and CNE2 cells, which have been proved to possess different EGFR expression levels, to validate our conjecture. The two-drug regimen showed a significant synergistic effect in HNE1 cells but an additive effect in CNE2 cells. Our results showed that cisplatin-induced apoptosis was significantly enhanced by cetuximab in the high EGFR-expressing HNE1 cells but not in CNE2 cells. Further molecular mechanism study indicated that the EGFR/AKT pathway may play an important role in cell apoptosis via the mitochondrial-mediated intrinsic pathway and lead to the different antitumor effects of this two-drug regimen between HNE1 and CNE2 cells. Thus, the regimen may be applied in personalized NPC treatments.

## 1. Introduction

Nasopharyngeal carcinoma (NPC) is a cancer arising from the nasopharynx epithelium. EBV infection has been proven to be the most relative and widely studied aetiological factor. NPC, particularly the classical nonkeratinizing type, is uncommon compared with other cancers worldwide, and it has a unique pattern of geographic and ethnic distribution which differs from other head and neck epithelial tumors. Most new cases occurred in southeast Asia and it is also endemic in southern China. Almost half of new cases present at an advanced stage. The role of surgery is limited because of its silent deep-seated location and anatomical proximity to critical structures. Fortunately, this cancer is highly radiosensitive and chemosensitive. Advances in management over the past three decades have dramatically improved overall prognosis. Current therapeutic strategies are based on disease stage [1]. Intensity-modulated radiotherapy (IMRT), a standard treatment for NPC, plays a preferred role in the treatment of patients with NPC. Excellent locoregional control can be achieved by the complete coverage of tumor targets while sparing critical normal structures [2]. Early studies reported 5-year local control rates ≥90% for T3 stage and 74%–80% for T4 disease [3–7]. A retrospective study of 1593 patients also showed absolute advantages in overall survival and disease-specific survival for IMRT [8]. Besides, the combination of chemotherapy and radiotherapy is another symbolical advancement in the treatment of NPC, for which cisplatin is the common basic chemotherapeutic drug. After the publication of the seminal INT-0099 trial, several trials have reported excellent advantages of cisplatin-based concurrent chemoradiotherapy in NPC management with a 5-year local control rate over 90%. Concurrent chemoradiotherapy is recommended as the standard treatment strategy in stage II-IVB NPC [9]. However, local recurrence and/or distant metastasis still confuse clinicians as the major pattern of disease failure. Therapy resistance, especially the cisplatin resistance, is the main cause of disease failure. Hence, a new potent systemic management is urgent for this cancer.

With the development of molecular-targeted therapy, epidermal growth factor receptor (EGFR) represents a promising new therapeutic target in various cancers. EGFR is proved overexpressed in approximately 85% of NPC and is involved in chemo/radioresistance and poor prognosis [10].

Cetuximab (C225), an anti-EGFR monoclonal humanized antibody interacting with the extracellular binding site of EGFR to block ligand stimulation, serves as a targeted therapy approved for the treatment of head and neck squamous cell carcinoma (HNSCC) [11]. The antitumor effect of C225 was studied in various human NPC cell lines (CNE-2, C666-1, HONE-1, and HK1) either alone or in combination with conventional cytotoxic drugs, such as cisplatin and paclitaxel. Sung et al. demonstrated that C225 showed a significant single agent antitumor effect and an additive effect with cisplatin or paclitaxel in NPC cell lines with high EGFR protein expression (HK-1 and HONE-1) but a minimal activity in NPC cell lines with a low expression (CNE-2 and C666-1) [12]. In addition, C225 enhanced the antitumor activity of several chemotherapeutic drugs in mouse xenograft models. Our study aims to elucidate the mechanism responsible for the combined effects in NPC cell lines.

## 2. Materials and Methods

### 2.1. Cell Lines and Cell Culture

The two NPC cell lines, HNE1 and CNE2, were provided by the Research Center of Clinical Oncology of the Affiliated Jiangsu Cancer Hospital (Nanjing Medical University, Nanjing, Jiangsu, China) and both originated from poorly differentiated human nasopharyngeal squamous cell carcinoma tissues. Both cells were maintained as previously described [13]. Both cell lines were cultured in Roswell Park Memorial Institute-1640 medium (Corning, Manassas, VA, USA) supplemented with 10% fetal bovine serum (FBS, Gibco, Grand Island, USA) at a 37°C humidified atmosphere containing 5% CO_2_.

### 2.2. Cell Viability Assay

Cell viability was quantified using a Cell Counting Kit-8 (CCK8, Dojindo, Kumamoto, Japan). Cells were cultured at a density of 5 × 10^3^ cells per well in flat bottomed 96-well plates. After 24 h of incubation at 37°C in 5% CO_2_, 200 *μ*L of cetuximab (MERCK) (62.5, 125, 250, 500, 1000, or 2000 *µ*g/mL) and/or cisplatin (Sigma) (0.25, 0.5, 1, 2, 4, or 8 *µ*g/mL) diluted with the medium to various concentrations were added to each well. After being incubated for 48 h, 10 *μ*L of CCK-8 was added to each well in accordance with the manufacturer's instructions. After 2 h, cell viability was quantified via reading the absorbance at 450 nm by using a SpectraMax 190 microplate reader (Molecular Devices, Sunnyvale, CA, USA). The percentage growth inhibition was calculated as (OD_control_ − OD_drug_)/OD_control_ × 100. The half maximal inhibitory concentration (IC50) values were calculated using GraphPad Prism 6.0 (GraphPad Software, San Diego, CA, USA). Based on the results, we chose optimal concentrations to continue our experiment. The experiment was performed in triplicate and repeated at least three times.

### 2.3. Calculation of Combination Index

The type of interaction between cisplatin and cetuximab was evaluated by comparing the cytotoxic effects obtained after simultaneous exposures to the drugs. The combination index (CI) was calculated using the following equation [14]: CI = CDDPc/C225e + C225c/CDDPe, where CDDPe and C225e are the concentrations of CDDP and C225 that inhibit *X*% of the proliferation when used alone and CDDPc and C225c are the concentrations of CDDP and C225 that produce the same effect when used in combination, respectively. With this method, drug synergism was analyzed as follows [15]: CI < 1 indicates a synergistic effect, CI = 1 an additive effect, and CI > 1 an antagonistic effect.

### 2.4. Quantitative Reverse Transcription PCR (qRT-PCR)

Total RNA was extracted from CNE2 and HNE1 cells using TRIzol reagent (Invitrogen, Carlsbad, USA) in accordance with the manufacturer's instructions. Total RNA (1 *µ*g) was reverse-transcribed using PrimeScript First-Strand cDNA Synthesis Kit (Takara, Dalian, China). qRT-PCR was performed using SYBR Green PCR Master Mix (Applied Bio-Systems, Carlsbad, CA, USA) on ABI7300 (Applied Bio-Systems). The primers for the qRT-PCR detection of EGFR mRNA (forward: 5′-GCCCCCACTGCGTCAAGACC-3′; reverse: 5′-ACCTGGCCCAGTGCATCCGT-3′) and actin (forward: 5′-TTCTACAATGAGCTGCGTCTG-3′; reverse: 5′-CAGCC TGGATAGCAACGTATC-3′) were synthesized by Invitrogen. Cycling parameters were followed in accordance with the protocol. All reactions were repeated three times for each sample. Primer quality was analyzed from dissociation curves. The fold change was determined as 2^−ΔΔCt^, where Ct is the fractional cycle number at which the florescence of each sample passes the field threshold. ΔCt was calculated by subtracting the Ct of actin from the Ct of the mRNA of interest. ΔΔCt was calculated by subtracting the ΔCt of the reference sample from the ΔCt of each sample.

### 2.5. Colony Formation Assay

Clonogenic survival assays were actualized as previously described. In short, the cells were harvested with trypsin-EDTA, counted, and then suspended in 1640 medium containing 10% FBS. The stock solution containing 50000 cells/mL was used, and 10 *µ*L of this solution was diluted in 2 mL of growth medium. The cells were treated with 0.5 *µ*g/mL cisplatin and 125 *µ*g/mL cetuximab on the basis of previous experiments. Finally, the plates were incubated at +37°C for 14 days with the drugs present throughout the entire incubation period. After treatment, the cells were stained with Giemsa and photographed. The experiments were performed three times.

### 2.6. Flow Cytometry

CNE-2 and HNE-1 cells were seeded at a density of 1 × 10^5^ cells per well in flat bottomed six-well plates. After 24 h of incubation, cells were treated with 2 mL of cetuximab (250 *µ*g/mL), cisplatin (1 *µ*g/mL), or the drug combination diluted with the medium to appropriate concentrations on the basis of the preexperiment results. The cultures were incubated for 48 h at 37°C in 5% CO_2_, and then the adherent and nonadherent cell fractions were harvested and washed twice with ice-cold phosphate buffered saline (PBS). After washing, the cells were resuspended in 100 *μ*L of binding buffer and then stained with 5 *μ*L of Annexin V-fluorescein isothiocyanate (FITC) and propidium iodide (PI) for 30 min at 4°C in the dark. Subsequently, 400 *μ*L of binding buffer was added to the mixture in accordance with the manufacturer's instructions (Nanjing KeyGen Biotech Co., Nanjing, China). The cells were immediately analyzed with a flow cytometer (EPICS XL, Beckman Coulter Inc., Fullerton, CA, USA). Early apoptotic cells stained positive for Annexin V-FITC and negative for PI. Late apoptotic cells were positive for both Annexin V-FITC and PI. The experiment was performed in triplicate and repeated at least three times.

### 2.7. Cell Cycle Analysis

Cells were treated with the same process as mentioned above. The adherent cell fractions were then trypsinized and fixed with 70% ice-cold ethanol overnight at −20°C. Cells were washed with ice-cold PBS and then stained with a solution of PI and RNaseA for 30 min at 37°C in the dark. Cell cycle distribution was analyzed using flow cytometry (EPICS XL, Beckman Coulter Inc., Fullerton, CA, USA). The data were analyzed using ModFit TM for Mac version 3.0 software (Verity Software House, Topsham, ME, USA). The experiment was performed in triplicate and repeated at least three times.

### 2.8. Western Blot Analysis

Cells were treated with the same process as mentioned above. Total cell proteins were extracted after treatment with cetuximab, cisplatin, or the combination of the drugs for 48 h. The cells were washed with PBS and lysed in Radio-Immunoprecipitation Assay buffer with phosphatase and protease inhibitors (Roche Diagnostics, Mannheim, Germany). The protein concentrations were determined using the Bradford protein assay kit (Bio-Rad Laboratories Inc., Hercules, CA, USA) in accordance with the manufacturer's instructions. Equivalent amounts of protein samples were separated by 10% sodium dodecyl sulfate-polyacrylamide gel electrophoresis and transferred onto polyvinylidene difluoride membranes (Millipore, Billerica, MA, USA). After blocking the membrane's nonspecific binding sites using nonfat milk, the membranes were separately incubated with specific primary antibodies overnight at 4°C, washed with PBS containing 0.1% (v/v) Tween 20, and then probed with HRP-conjugated goat antirabbit secondary antibodies. The specific proteins of interest were visualized using the enhanced chemiluminescence Western blot detection reagents. Densitometric quantification of the bands was performed using the Bio Image Intelligent Quantifir 1-D (Version 2.2.1, Nicon-BioImage Ltd., Japan). The target proteins were detected with primary antibodies recognizing EGFR, p-EGFR, AKT, p-AKT, caspase-3, cleaved caspase-3, and Bax (Cell Signaling Technology). *β*-actin was used as a loading control. The experiment was performed in triplicate and repeated at least three times.

### 2.9. Statistical Analysis

All values are expressed as means ± standard errors of means (SEM) of at least three independent experiments. Statistical analysis was performed with GraphPad Prism 6.0 (GraphPad Software, San Diego, CA, USA). For in vitro assays, statistical significance was reported if the *p* value was <0.05 using an unpaired Student's *t*-test.

## 3. Results

### 3.1. Cetuximab Enhanced Cisplatin-Induced Cytotoxicity in NPC Cells and a Moderate Synergistic Effect Was Observed in HNE1 Cells

**Figure 1:**
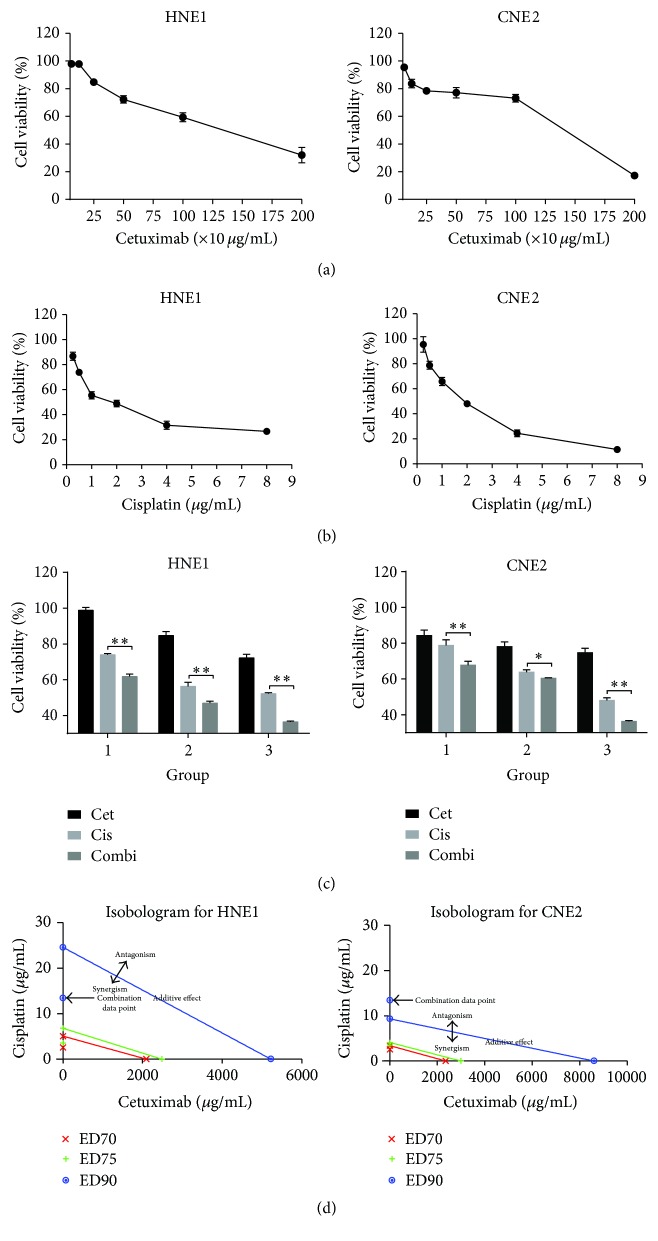
Effects of cisplatin, cetuximab, and a combination of both on NPC cell growth. HNE1 and CNE2 cells were treated with the indicated concentrations of (a) cetuximab, (b) cisplatin, or (c) 1 *µ*g/mL cisplatin and 250 *µ*g/mL cetuximab for 48 h, and cell viability was determined by a CCK-8 assay. (d) The classic isobologram of HNE1 and CNE2 cells. ^*∗*^ *p* < 0.05, ^*∗∗*^ *p* < 0.01 versus cisplatin treatment alone.

We performed a CCK-8 assay to explore the antitumor effects of cetuximab and cisplatin administered alone or in combination on CNE2 and HNE1 cells. Cells were plated and incubated with various concentrations of cisplatin (up to 8 *µ*g/mL) with or without cetuximab (up to 2000 *µ*g/mL) for 48 h. Cell viability was determined using a 96-well CCK8-based colorimetric assay. As shown in Figure 1, both CNE2 and HNE1 cells incubated with progressive concentrations of cetuximab only showed low cell cytotoxicity, 1045 (95%, (483, 2260)) and 1182 *µ*g/mL (95%, (688, 2030)), respectively. By contrast, the cells treated with cisplatin alone showed a significant dose-based activity. The corresponding IC50 values of cisplatin on CNE2 and HNE1 cells were 1.801 ± 0.551 and 1.875 ± 0.608 *µ*g/mL, respectively. Treatment with double agents in these two cell lines could achieve an additive cytotoxic effect compared with the single agent groups (*p* < 0.05). As previously mentioned, treatment of HNE1 cells with cetuximab and cisplatin concurrently increased cell growth inhibition compared with the single agent groups. The combination index (CI) was calculated using the equation mentioned above (Table 1). The CIs at IC75 of CNE2 and HNE1 cells were 0.9 and 0.5, respectively. An isobologram was drawn by Calcusyn Software based on the Chou-Talalay method, which can distinguish between the synergistic and additive effects of two compounds with the constant ratio combination design, confirming that the combination of cetuximab and cisplatin resulted in a remarkable synergistic growth inhibitory effect on HNE1 (Figure 1(d)). In contrast, the combined treatment exhibited an additive or moderate synergistic effect in the CNE2 cells.

**Figure 2:**
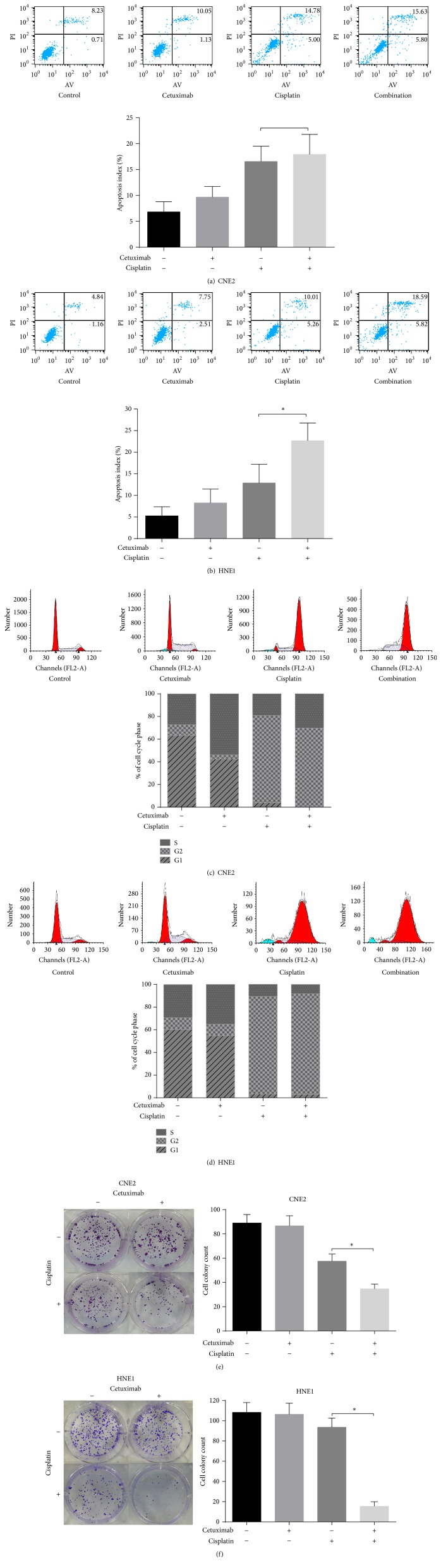
Cetuximab enhanced cisplatin-induced apoptosis and antiproliferation effect in HNE1 cells. HNE1 cells were treated with cetuximab, cisplatin, or the double agents for 48 h before the cells were analysed by flow cytometry ((a), (b), (c), and (d)). Multiplication capacity of cells in different treatment groups is shown in ((e), (f)) via a colony formation assay. ^*∗*^ *p* < 0.05 versus cisplatin treatment alone.

Subsequently, we conducted a colony formation assay to detect the concurrent effect of cetuximab and cisplatin on the proliferative capacity of these two cell lines. The six-well plate colony formation assay was described above in detail. After treatment for 14 days, cells were photographed using an inverted phase-contrast microscope (Figure 2(e)). The number of individual colonies in the combination group was visibly less than that in the single agent groups.

Cisplatin (1 *µ*g/mL) and cetuximab (250 *µ*g/mL) were used for subsequent experiments because these concentrations were less than IC50 with a low single agent cytotoxicity.

### 3.2. Cetuximab Enhanced Cisplatin-Induced Apoptosis in HNE1 Cells

Flow cytometry was conducted to assess whether or not the growth inhibitory effect is mediated through the enhancement of cisplatin-induced cell apoptosis. The rate of apoptosis in both cells increased after treatment with the combination of cisplatin and cetuximab compared with both single agents (Figures 2(a) and 2(b)). This difference was statistically significant in HNE1 (*p* < 0.05) but not in CNE2. To identify the mechanism, we examined the effect on cell cycle arrest. Finally, we observed a significant level of G2/M phase arrest in both cell lines after treatment with cisplatin in the absence or presence of cetuximab when compared with the control or cetuximab group, but no significant difference was found between the double and single agent groups (Figures 2(c) and 2(d)). These results suggest that the growth inhibitory effect is mediated through the enhancement of cisplatin-induced apoptosis but not cell cycle arrest.

### 3.3. EGFR mRNA Expressed Discriminatively in These Two Cell Lines

**Figure 3:**
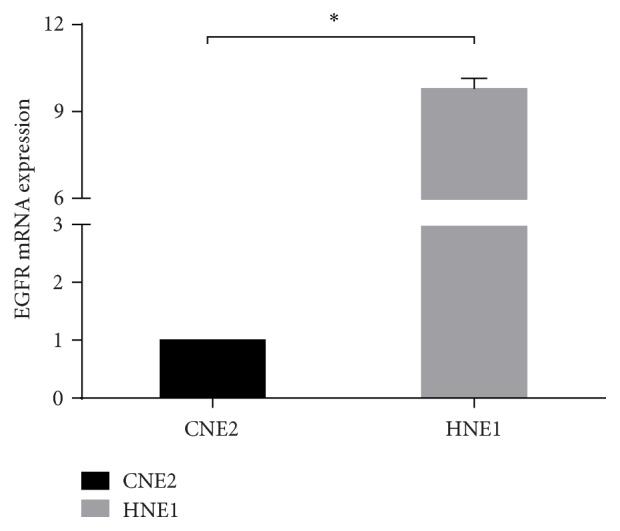
Relative levels of EGFR mRNA expression in NPC cell lines (CNE2, HNE1), as determined by qRT-PCR analysis. ^*∗*^ *p* < 0.05 versus CNE2.

The mRNA expression levels of EGFR in the CNE2 and HNE1 cell lines were detected via qRT-PCR analysis. The results are shown in Figure 3. The expression level of EGFR mRNA was over 9-fold in HNE1 compared to that in CNE2 cells, and the difference was statistically significant.

### 3.4. Effects of the Combination Treatment of Cetuximab and Cisplatin on the EGFR and AKT Signaling Pathways

**Figure 4:**
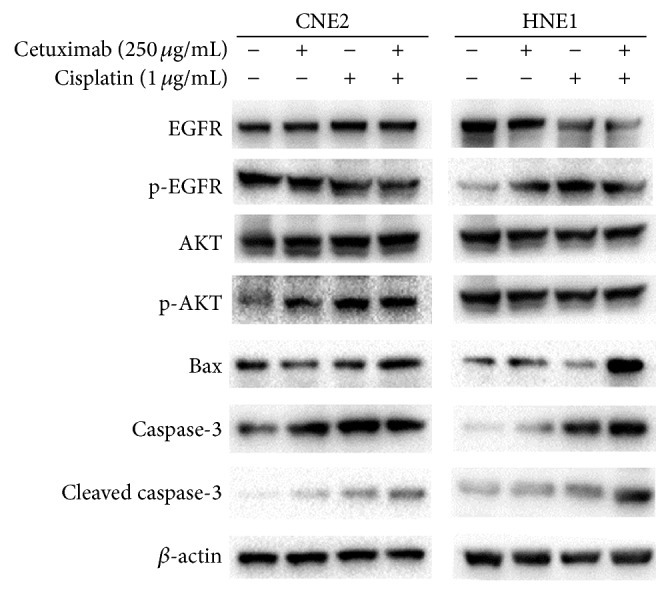
Effects of combination treatment with cetuximab and cisplatin on the expressions of proapoptosis proteins and EGFR/AKT signaling pathway proteins. Cells were treated with cetuximab, cisplatin, or the combination treatment of cetuximab and cisplatin for 48 h. The expressions of EGFR, p-EGFR, AKT, p-AKT, Bax, caspase-3, and cleaved caspase-3 in both cells were detected by Western blotting analysis. *β*-actin was used as a loading control.

To characterize EGFR downstream signaling that may correlate with the synergistic inhibitory effects of cetuximab and cisplatin on NPC cells, the levels of EGFR and its downstream signaling pathway protein were analyzed via Western blot. Interestingly, cisplatin/cetuximab combination suppressed EGFR phosphorylation and total EGFR in HNE1 cells but not in CNE2 cells (Figure 4). Furthermore, we found that the expression of p-AKT was upregulated by cisplatin treatment in both cells. In addition, treatment with a combination of both agents decreased p-EGFR and p-AKT expression in HNE1 cells.

### 3.5. Effects of the Combination Treatment of Cetuximab and Cisplatin on Bax, Caspase-3, and Cleaved Caspase-3

We examined the expression of Bax, caspase-3, and cleaved caspase-3 protein via Western blot analysis to confirm that apoptosis occurred in response to a combination of cetuximab and cisplatin. As shown in Figure 4, the expression of these proapoptosis proteins was higher in the combination groups than in the single agent groups in HNE1 cells. Our results showed that cetuximab increased cisplatin-induced apoptosis in HNE1 cells.

## 4. Discussion

NPC is distinguished from other types of head and neck cancers due to its unique sensitivity to both radiotherapy and chemotherapy. In spite of the employment of cisplatin-based chemoradiotherapy, the treatment outcome for advanced stage is still unsatisfactory because of local recurrence and/or distant metastasis as the major pattern of disease failure. As the basal chemotherapy drug for NPC management, cisplatin has a dose limitation due to its high nephrotoxicity. Hence, a new potent systemic therapy is urgent for this cancer.

Cisplatin, a DNA-damaging agent, is a classical chemotherapeutic employed for the treatment of various human cancers. Although cisplatin-based concurrent chemoradiotherapy has been a symbolical standard strategy in the management of locally advanced NPC, distant failure still confuses clinicians with a distant relapse rate as high as 34% and the outcome of metastatic NPC remains disappointing [1, 16]. Previous studies have examined that the combination of cetuximab and cytotoxic agents can enhance the inhibitory effect both in vitro and in vivo [11]. Nevertheless, the molecular mechanism remains to be clarified.

In our study, we investigated the combination efficacy of cetuximab and cisplatin on anticancer and its molecular mechanism in human NPC lines HNE1 and CNE2. Our results demonstrated that the combination treatment of cetuximab and cisplatin at a concentration lower than IC50 showed a synergism effect on cell growth and apoptosis in HNE1 cells but an additive effect in CNE2 cells. Present in vitro and clinical studies have shown that the EGFR protein is overexpressed in NPC, although it varies among different NPC cell lines. Further studies demonstrated that cetuximab selectively produces dose-dependent single agent cytotoxicity in certain NPC cell lines only, which might correlate with the level of EGFR protein expression [12]. To explore the potential cause of this different action, we detected the mRNA expression levels of EGFR in the CNE2 and HNE1 cell lines and further demonstrated that these two cell lines had differential expression of EGFR mRNA with a higher expression in HNE1 cells. Therefore, we conjectured that cetuximab and cisplatin might show different combination effects in human NPC cell lines HNE1 and CNE2 which were different in the expression level of EGFR. Such differential effect has been reported in esophageal squamous cell carcinoma previously [17]. Kwon et al. reported that combination of cetuximab and cisplatin resulted in a growth inhibition only in the EGFR overexpressed TE-8 cell line. Furthermore, they confirmed that cetuximab inhibited cisplatin-induced EGFR activation in TE-8 but not in TE-4 cells.

Most cytotoxic chemotherapeutic drugs cause apoptosis in cancer cells. We demonstrated that the apoptosis rate of the combination group was significantly increased as compared with cetuximab or cisplatin treatment alone. To determine the mechanism underlying cetuximab enhanced cisplatin-induced apoptosis in HNE1 cell line, the total and phosphorylation expression status of EGFR and AKT were examined in both cells by Western blotting. Expression of p-EGFR and p-AKT was upregulated by cisplatin treatment in HNE1 cells which was higher in EGFR expression, indicating an activation of EGFR signaling. Interestingly, the cisplatin-induced increases in p-EGFR and p-AKT expression in HNE1 cells were abrogated in the presence of cetuximab. In CNE2 cells, treatment with a combination of both agents led to a decrease in p-EGFR expression but had no effect on the expression or phosphorylation of AKT. Additionally, in HNE1 cells, we found that Bax and the cleavage of caspase-3 protein were dramatically increased by the combination treatment when compared with cetuximab or cisplatin treatment alone. The results of the present study, showing that the combined effects are more significantly observed in NPC cell lines with higher overexpressing EGFR, support the phenomenon that treatment with a common protocol demonstrated different efficacy and outcome among patients. PI3K/AKT/mTOR, JAK/STAT, and Ras/Raf/MAPK pathways are well known to be involved in activation of EGFR cell signaling cascades [18]. About 200 targets of EGFR signaling pathway have been reported, and 177 molecules involved in EGFR signaling pathway are listed in the Human Protein Reference Database (http://www.hprd.org/), but EGFR signaling pathway in NPC still remains to be elucidated [19]. Ligand-independent EGFR activation acts as a survival signal in squamous cell carcinoma that can trigger phosphatidylinositol 3′-kinase- (PI3K-) mediated AKT activation [20, 21]. PI3K/AKT, a well-known important signaling pathway in NPC, has been reported to be closely related to therapeutic resistance [22, 23]. Dysregulation of phosphatidylinositol-3- kinase/protein kinase B (PI3K/AKT) is associated with the deficiency of apoptosis and the phenotype of multidrug resistance in cancer cells [24, 25]. The EGFR/PI3K/AKT signaling pathway has been proven to be associated with the ADAM17-mediated cisplatin resistance of hepatocellular carcinoma cells [26]. Overall, we conjecture that similar activation might exist in NPC cells, and interfering it might be a useful approach to sensitize these cells to cisplatin. In this study, we demonstrated that cetuximab might enhance cisplatin-induced cell apoptosis in HNE1 cells, as was high EGFR expression relatively, via inhibition of cisplatin-enhanced EGFR/AKT activation. However, earlier Kramer's work demonstrates that EGFR-mediated survival effects were primarily through activation of ERK, but not AKT [27]. Besides, Son et al. also demonstrated synergistic inhibitory effects of cetuximab and cisplatin on human colon cancer cell growth via inhibition of the ERK-dependent EGF receptor signaling pathway [28]. Thus, we will further investigate the impact on EGFR/ERK signaling of the combination with cetuximab and cisplatin in NPC cells, to make our work consummate.

Cell cycle is also a main regulatory mechanism of cell growth, and many chemical compounds could trigger apoptosis in cancer cells accompanied by cell cycle arrest. In our study, we examined the effect of these two drugs on cell cycle arrest. As mentioned above, cisplatin treatment in the presence or absence of cetuximab in HNE1 and CNE2 cells induced G2/M phase arrest compared with cetuximab treatment alone. However, no significant difference was found between the combination treatment group and the single agent groups in both cell lines. Thus, we can infer that the synergistic enhancement of the antitumor effect induced by the combination treatment of cetuximab and cisplatin in HNE1 cells occurs with the increase in cisplatin-induced apoptosis.

## 5. Conclusions

In summary, our study observed a synergistic effect of the combination of cetuximab and cisplatin in NPC cells. The results are consistent with our conjecture and previous reported data in esophageal squamous cell carcinoma [12, 17]. Interestingly, the two-drug regimen of cetuximab and cisplatin showed different combination effects between HNE1 and CNE2 cells, which might be related to the different inhibition of the EGFR/AKT pathway and the mitochondrial-mediated cell apoptosis pathway. We predict that the combination therapy of cetuximab and cisplatin may contribute to different survival outcomes for patients depending on the presence of EGFR gene amplifications, which can be applied toward personalized NPC treatments. The results of our study may help in the development of personalized and effective treatment regimens that are tailored to individual patients. However, the efficacy of cetuximab combined with cisplatin in clinical practice for NPC remains in dispute and looks forward to the results of prospective, randomized controlled trials.

## Figures and Tables

**Table 1:** The combination indexes (CIs) of cetuximab and cisplatin in CNE2 and HNE1 cells.

	Combination index values at		
	IC50	IC70	IC75
CNE2	0.9	0.8	0.9
HNE1	0.6	0.5	0.5

IC50: half maximal inhibitory concentration (IC50) values. IC70: 70% inhibitory concentration values. IC75: 75% inhibitory concentration values. CI: combination index.
